# A Perspective on the 6th International Conference on Sports Concussion

**DOI:** 10.3390/brainsci14050515

**Published:** 2024-05-20

**Authors:** Haruo Nakayama, Yu Hiramoto, Satoshi Iwabuchi

**Affiliations:** Department of Neurosurgery, Toho University Ohashi Medical Center, Tokyo 153-8515, Japan; yu.hiramoto@med.toho-u.ac.jp (Y.H.); iwabuchi@med.toho-u.ac.jp (S.I.)

**Keywords:** sports-related concussion, Amsterdam statement, International Concussion in Sport Group

## Abstract

The International Conference on Sports Concussion, held every four years since 2001, has been instrumental in forming the international consensus on sports-related concussions. However, due to the unprecedented global pandemic of COVID-19, not only the Tokyo Olympics 2020, but also the initially scheduled sixth conference was postponed multiple times. Finally, the 6th International Conference on Sports Concussion took place in Amsterdam at the end of October 2022. In July 2023, the Amsterdam Declaration, reflecting the outcomes of this sixth conference, was released. This paper provides an overview of the conference, in which significant updates were revealed and introduced, including revisions to the definition of sports-related concussions, as well as the latest version of the Sports Concussion Assessment Tool (SCAT), known as SCAT6, the Office Assessment Tool (SCOAT), and the updated staged return-to-play protocol.

## 1. Introduction

The International Consensus Statement on Concussion in Sport, known as the “Amsterdam Statement”, was developed for healthcare providers (HCPs) involved in the management and support of athletes at risk of, or suspected to have suffered, sports-related concussions (SRCs) across all levels of sport, from recreational to professional [1]. The statement is not intended to be used as a prescriptive guideline, but rather as a set of flexible recommendations that can be adapted to a wide range of sporting, clinical, and cultural environments, because adherence to its principles must consider differences in geography, medical infrastructure, and culture. Moreover, as it primarily presents recommendations and a summary of the consensus process, HCPs are encouraged to read the full details of the process, the ten systematic reviews that influenced its outcomes, and associated methodological papers to deepen their understanding of the subject.

## 2. The International Concussion in Sport Group

The first international conference of the International Concussion in Sport Group (CISG) was held in Vienna in 2001 [2]. The CISG was initially established with cooperation between the International Olympic Committee (IOC), the International Federation of Association Football (FIFA), and the International Ice Hockey Federation (IIHF). Following its inaugural meeting in Vienna, the CISG released the world’s first consensus statement on SRCs, a publication that summarized the global expert agreement on the subject. 

In recent years, just as with the Summer Olympics, the conference has been held in the fall of the host year, with the second (Prague, 2004), third (Zurich, 2008), fourth (Zurich, 2012), and fifth in Berlin in 2016. The consensus statement published thereafter, known as the “Berlin Statement”, provided a logical framework/sequence for SRC management along with points of discussion in the form of 11 “Rs”: “*Recognize*, *Reduce*, *Remove*, *Refer*, *Re-evaluate*, *Rest*, *Rehabilitation*, *Recover*, *Return-to-Learn (RTL)/Return-to-Sport (RTS)*, *Reconsider*, and *Residual Effects*” [3]. The Amsterdam Statement builds on the framework of its predecessor with two additional “Rs”: *Retire*, which introduces considerations for retirement or discontinuation from sports after SRC; and *Refine*, which highlights “the need to embrace ongoing strategies to advance the field” along with “several considerations that could strengthen the consensus process”.

## 3. Purpose of the Amsterdam Statement

The stated objectives of the Amsterdam Statement are “to update current recommendations for SRC through an evidence-informed consensus methodology”, and “to provide a summary of evidence-based and practice recommendations based on the consensus of science and expert panel at the time of the conference”. During the conference, consensus agreement was determined through anonymous electronic voting by expert panel members, and a recommendation was deemed to have achieved consensus if 80% or higher agreement was achieved [4]. The conference also produced “freely available evidence-informed tools to assist in the detection and assessment of SRC”, including the Concussion Recognition Tool-6 (CRT6), the Sport Concussion Assessment Tool-6 (SCAT6), the Child SCAT6, the Sport Concussion Office Assessment Tool-6 (SCOAT6), and the Child SCOAT6 [5,6,7,8,9]. 

## 4. Definition of Sports-Related Concussion

The definition of SRC from the Amsterdam Statement is reprinted below [1]. Rreproduced with permission from BMJ Publishing Group Ltd.

“*An SRC is a traumatic brain injury (TBI) caused by a direct blow to the head, neck, or body that occurs in sports and exercise-related activities, resulting in an impulsive force being transmitted to the brain which initiates a neurotransmitter and metabolic cascade possibly causing axonal injury, changes in blood flow, and inflammation affecting the brain. Symptoms and signs may present immediately, or evolve over minutes or hours, which commonly resolve within days, but may be prolonged.*

*No abnormality is seen in standard structural neuroimaging studies, such as computed tomography or magnetic resonance imaging T1- and T2-weighted images, but in research settings, abnormalities may be present in functional, blood flow, or metabolic imaging studies. SRCs can cause a wide range of fascinating clinical symptoms and signs, which may or may not involve loss of consciousness. The clinical symptoms and signs of concussion are not solely explained by drug, alcohol, or medication use; other injuries, such as cervical injuries and peripheral vestibular dysfunction; or other comorbidities, such as psychological factors or coexisting medical conditions.*”

The first sentence is consistent with the definition of SRC outlined in the Berlin Statement, but the full text has been improved by integrating and summarizing the latest research findings on its pathophysiology, neuroimaging diagnostics, and clinical symptoms reported since its publication. Notwithstanding these enhancements, approaching this definition with caution remains important, as, like all previous CISG consensus statements, it contains many conceptual elements, while lacking specific diagnostic criteria. Furthermore, the statement also acknowledges that even the conceptual definition above did not reach consensus at the sixth CISG conference, given that the American Congress of Rehabilitation Medicine (ACRM) recently revised the definition of mild TBI (mTBI) for the first time in 30 years [10]. A topic for future consideration is the development of a single conceptual and operational definition.

## 5. CRT6, SCAT6, and SCOAT6

The SCAT (Sport Concussion Assessment Tool) was first compiled as an international tool for the evaluation of concussions at the second CISG conference (Prague, 2004) [11]. The Pocket SCAT2 was introduced at the third CISG conference (Zurich, 2008) with the goal of making the SCAT accessible to non-medical personnel [12]. At the fourth conference (Zurich, 2012), the SCAT3 and its pediatric counterpart, the Child SCAT3, were published [13], and the Pocket SCAT evolved into the CRT (Concussion Recognition Tool). The SCAT5, the Child SCAT5, and the CRT5 were released in tandem with the Berlin Statement after the fifth conference (Berlin, 2016) [3]. The sixth conference in Amsterdam produced the newest revision of the SCAT (SCAT6), along with a new instrument, the SCOAT6, recommended in the Amsterdam Statement to be used in sideline assessments and office assessments, respectively [5,7,14].

**Figure 1:**
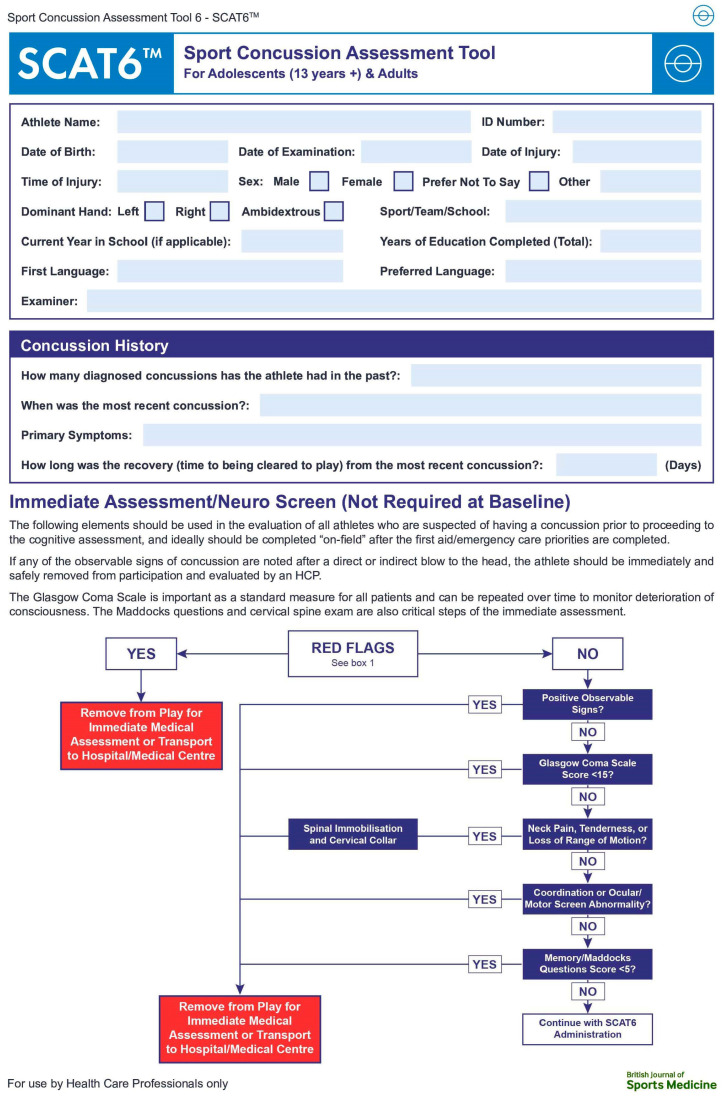
Cascade of immediate assessment [16]. Reproduced with permission from BMJ Publishing Group Ltd.

The Amsterdam Statement introduces the latest versions of SRC assessment tools of the CISG, including the CRT6 [8,9], the SCAT6 [14], and the Child SCAT6 [15]. These tools have been optimized for evaluation in the acute phase, ideally within the first 72 h following SRC, and up to a week at most [1]. The SCAT6, intended for use in clinical settings, now features a cascade diagram at its outset, which shows users what to do next based on each assessment and its results after a head injury or a suspected SRC event occurs (Figure 1) [16]—a feature absent from the SCAT5 [16]. Conversely, the SCOAT6 and the Child SCOAT6 have been designed to provide better guidance for evaluation and management in office settings after 72 h post-injury, as well as for continuous assessment over the following weeks [5,6,7]. The SCAT6 and the SCOAT6 have been designed with intentional overlap to facilitate transitions between these tools. Finally, in acknowledgement of the scarcity of validated data for children compared with adults, the age range has been updated from 5–12 years in the Child SCAT5 [17] to 8–12 years in the Child SCAT6 [15].

The CRT6 was developed for use by non-medical professionals, on the basis that medical personnel who have received proper training are unlikely to be present on the sidelines at youth sporting events, and the statement strongly recommends that it be used by all adults who supervise sports for children and young people [8,9]. Further educating adults involved in youth sports about the CRT remains a significant challenge, given that in Japan, as well as Western countries, medically trained personnel are hardly a constant presence at youth sporting events.

The CISG has issued official rationales for regulating the translation of the CRT6, the SCAT6, and the SCOAT6 into languages other than English. Although not directly mentioned in the Amsterdam Statement, they are published on the organization’s website [18], and reprinted below for convenience.

“*Adaptation of the instruments requires expertise in the target language and culture and consultation with a neuropsychologist or linguist experienced in the process of cultural adaptation of psychological/neuropsychological instruments. Lists of English words have been developed using words with the same level of complexity and familiarity in the target language. This process must be replicated in any cultural adaptation. Although differences exist across language groups, the recommended approach is to select a pool of words with the same level of complexity (e.g., word length, phonemic/tonal complexity), similar frequency of use in the target language (word frequency tables are widely available), and at approximately the same developmental level, e.g., highly familiar with 8-year-old and above. Care must be taken to ensure that languages with many dialects either have separate lists by dialect or words are chosen such that they would be widely understood across dialects. Once developed, the lists should be pilot-tested on native language speakers and adjusted based on feedback received.*” 

The SCAT6 contains a large set of instructions to ensure it is properly administered, which underscores the importance of following a standardized assessment method to ensure the results obtained are accurate.

## 6. The Return-To-Sport Strategy

**Figure 2:**
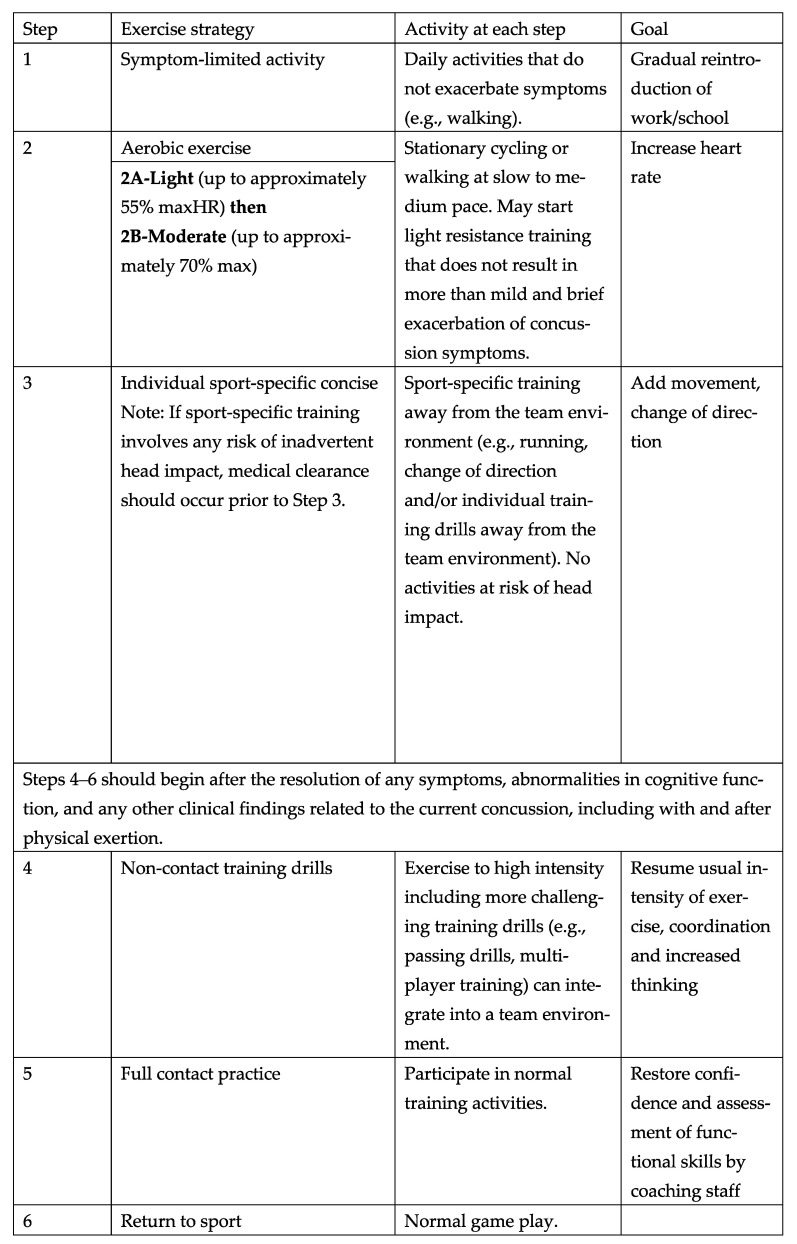
Return-to-sport (RTS) strategy. Each step typically takes a minimum of 24 h. Mild and brief presentation of symptoms, an increase of no more than 2 points on a 0–10 point scale for less than an hour when compared with the baseline value reported prior to physical activity. Athletes may begin Step 1 (i.e., symptom-limited activity) within 24 h of injury, with progression through each subsequent step typically taking a minimum of 24 h. If more than mild exacerbation of symptoms (more than 2 points on a 0–10 scale) occurs during Steps 1–3, the athlete should stop and attempt to exercise the next day. Athletes experiencing concussion-related symptoms during Steps 4–6 should return to Step 3 to establish full resolution of symptoms before engaging in activities at risk. A written determination of readiness to RTS should be provided by a HCP before unrestricted RTS as directed by local laws and/or sporting regulations [16]. Reproduced with permission from BMJ Publishing Group Ltd. HCP, healthcare professional; maxHR, predicted maximal heart rate according to age (i.e., 220-age).

Stepwise approaches have conventionally been recommended for RTS participation following a SRC. The “RTS Strategy” outlined in the Amsterdam Statement is an updated version of the “Graduated RTS Strategy” in the Berlin Statement. However, the principles that RTS should be conducted in conjunction with RTL and under the supervision of a qualified HCP remain unchanged. This latest RTS Strategy consists of six steps, divided into the first three steps and the last three steps (Figure 2) [1]. This demarcation is regarded as beneficial for the athlete and their support network (e.g., parents, coaches, administrators, and agents).

Step 1 (“symptom-limited activity”) takes place roughly 24–48 h after a concussion. This period of ‘relative’ rest is distinguished from traditional ‘absolute’ rest by allowing for activities that do not negatively affect activities of daily living. From 0 to 48 h post-SRC, relative (not strict) rest is recommended, which includes reducing screen time (e.g., watching television, playing video games, and using mobile phones). From 24 to 48 h post-SRC, athletes can return to light-intensity physical activity (PA), such as walking, provided it does not provoke or exacerbate symptoms.

Step 2 (“aerobic exercise”) is divided into two parts—light aerobic exercise (2A) and moderate aerobic exercise (2B)—which marks a significant change in the RTS strategy, effectively making it a seven-step process. HCPs who have access to exercise testing equipment can safely prescribe aerobic exercise within 2 to 10 days of a SRC, providing it does not exceed a heart rate threshold (HR_t_) that mildly exacerbates symptoms during testing. If SRC-related symptoms increase by no more than 2 points (10-point scale) compared with the pre-exercise baseline and last less than 1 h, the athlete can maintain or increase the duration and intensity of PA or the prescribed aerobic exercise. However, if symptoms worsen by more than 2 points or persist for over an hour, they should stop the PA, exercise, and cognitive exertion, resuming only once the symptoms have returned to previous levels. There is evidence that subthreshold aerobic exercise prescribed within 2–10 days of SRC is effective in both reducing the incidence of persistent symptoms after concussion (lasting more than one month) and in aiding the recovery in athletes whose symptoms last longer than one month [19].

For Step 3 (“individual sport-specific exercise”) to be completed, medical clearance must be obtained from a HCP before advancing to Step 4. This clearance is dependent on the full resolution of all SRC-related symptoms, cognitive abnormalities, and clinical findings in relation to the current concussion, including the absence of symptoms during and after physical exertion, and should be confirmed before the athlete is allowed to participate in practice involving physical contact. If practice activities in Step 3 involve all accidental exposure hazards, this clearance should be obtained from a HCP after completing Step 2 and before advancing to Step 3.

Each step in the RTS Strategy typically takes at least 24 h, as in previous versions. Clinicians and athletes should anticipate a minimum of one week to complete the full program, although unrestricted RTS typically takes up to one month post-SRC to achieve. As the RTS timeline differs from athlete to athlete, clinical management requires an individualized approach. If athletes have difficulty progressing through the RTS program, or their symptoms and signs do not gradually improve after the first 2–4 weeks, they may benefit from the involvement of a multidisciplinary team, including HCPs experienced in rehabilitation medicine and SRC management, alongside the RTL and RTS strategies. Before athletes return to activities with any risk of contact, collision, or falls (e.g., multi-player training drills), a medical determination of their readiness is strongly advised in the Amsterdam Statement. However, this decision is left to the discretion of HCPs in the Amsterdam Statement, in which neither specific items nor methods of clinical assessment are specified, marking a limitation of this recommendation.

## 7. Persisting Symptom Management

The Amsterdam Statement includes a revised definition of “persisting symptoms” of concussion, previously defined as those lasting over two weeks in adults and over four weeks in children, to include all symptoms that last over four weeks, regardless of whether in childhood, adolescence, or adulthood. In terms of specific recommendations, it suggests that persistent symptoms should be assessed using standardized and validated symptom rating scales because the various symptoms observed in concussion are non-specific and may be exacerbated by a variety of biopsychosocial factors other than concussion. However, the existing research has been considered insufficient to make evidence-based recommendations regarding the use of specific tests or measures in diagnosing persisting symptoms across all age groups [20]. Consequently, the Amsterdam Statement favors a multimodal clinical assessment approach to characterize patients with persisting symptoms, ideally conducted by a multidisciplinary team. Such a “SRC clinical network” may include sports doctors, athletic trainers/therapists, physiologists, physiotherapists, occupational therapists, sports chiropractors, neurologists, neurosurgeons, neuropsychologists, ophthalmologists, optometrists, rehabilitation physicians, psychologists, and psychiatrists. 

## 8. “Retire”

There were no recommendations related to retirement from contact or collision sports in the Berlin Statement or any previous versions, based on the lack of sufficient scientific evidence regarding retirement. Nonetheless, some sports and organizations have their own regulations regarding medical clearance for participation [21]. The Amsterdam Statement acknowledges that decisions are complex and multifaceted when it comes to retiring or discontinuing participation in contact or collision sports. Thus, physicians with expertise in TBI and sports should be involved, preferably a multidisciplinary team. Such decisions should be informed by a comprehensive clinical assessment that takes sociocultural factors into consideration. Importantly, HCPs should properly communicate with athletes and their families, including parents in the case of children and adolescents, regarding medical or scientific facts, uncertainties about their condition, and potential risks associated with returning to the chosen sport.

## 9. “Refine”

This section of the Amsterdam Statement is primarily focused on para sports and pediatrics, two areas that share the common limitation of insufficient research to inform evidence-based recommendations. Globally, an estimated 10% of children and adolescents have disabilities, but reports indicate a positive trend towards increasing opportunities for these individuals to participate in PA and sports. Disabilities that manifest in childhood, such as developmental disorders and cerebral palsy, inherently affect the function of the central nervous system (CNS), potentially leading to varied severity in concussion symptoms and differences in recovery timelines among affected individuals. Such differences in neurology between child para-athletes and athletes without disabilities against the backdrop of more participation in sports underscore the need for more research on concussions in child para-athletes, and experts have advocated for greater caution in their assessment and potential extended periods of rest post-SRC [22]. Furthermore, gradual return-to-play protocols should be customized to individual athletes’ needs, including modifications of assessments to accommodate specific disabilities, such as using an arm ergometer instead of a treadmill or stationary bike.

## 10. Limitations 

The Amsterdam Statement reflects the scientific evidence available at the time of the sixth CISG conference, but as concussion research continues to evolve, it will need to be updated to incorporate the latest scientific insights. It is vital to recognize that the statement is not intended to serve as a definitive set of clinical guidelines or legal care standards, but rather to aid HCPs as a suggested set of scientifically justified clinical practices. Every treatment should be tailored individually based on athletes’ specific symptoms and circumstances. Actually, although the statement does mention a definition of concussion, the lack of specific diagnostic criteria is its greatest weakness. In addition, the lack of evidence regarding the use of specific tests and measures in the diagnosis of persistent concussion symptoms is another weakness. In the future, it is hoped that these weaknesses can be overcome by establishing diagnostic methods and surrogate markers that can be used to assess the course of symptoms.

## 11. Conclusions

When managing concussions or athletes at risk in actual practice, HCPs should adapt and judiciously apply the recommendations in accordance with their unique environment, taking into account the specific cultural context of their country and region, their available medical and social resources, as well as healthcare infrastructure.
